# Is the ^18^F-FDG PET/CT the definite resource to detect the recurrence on high-risk thyroid cancer patients?

**DOI:** 10.1590/2359-3997000000300

**Published:** 2017-09-01

**Authors:** Carlos Alberto Buchpiguel

**Affiliations:** 1 Departamento de Radiologia e Oncologia Faculdade de Medicina Universidade de São Paulo São Paulo SP Brasil Departamento de Radiologia e Oncologia da Faculdade de Medicina da Universidade de São Paulo (FMUSP), São Paulo, SP, Brasil

Thyroid cancer is the most common endocrine neoplasm (1). Besides its good prognosis and indolent clinical course, more aggressive stages pose some challenges and may impair the morbidity/mortality rate in high-risk patients. So in those cases, is mandatory to optimize the diagnostic work-up in order to detect recurrences and metastases as early as possible for an effective therapeutic planning (2).

High-risk patients and undifferentiated tumors loose the capacity of trapping iodine-131, making not only the diagnosis but also the treatment of those patients a challenge. Even when conventional imaging is not able to localize the recurrence besides the rise of the serum thyroglobulin (Tg) levels, it is clinically valuable to pursue other alternatives to localize the sites of recurrent disease. Actually, neck ultrasound and chest computed tomography are efficient to detect the most common sites of recurrent thyroid tumor (3). However, it is not rare to see high-risk patients with elevated Tg and no signs of recurrence on conventional imaging, including iodine-131 whole body survey (WBS) (4).

PET-CT emerged as a molecular imaging tool, where the disease is detected more due to the molecular profile and/or metabolic cellular signaling than structural or functional abnormalities. The high rate of anaerobic glycolysis is one of the main features of various malignant tumors, and that is the reason for using fluordesoxyglucose labeled with fluoride-18 (FDG), a common positron emitter produced on Cyclotrons (5). Dedifferentiating thyroid tumors overexpress GLUT (glucose transporter proteins located on the cellular membrane) and also hexokinase-II (HK-II) that are the two major conditions for promoting and facilitating glucose uptake in the malignant cells. A reasonable number of publications are seen in the literature showing the value of FDG-PET in the evaluation of patients with thyroid carcinoma. A recent meta-analysis published by Haslerud and cols. (6) showed a pooled sensitivity and specificity of 79.4% for detecting recurrent well-differentiated thyroid carcinoma (WDTC). After the year 2000, the majority of PET scanners were shipped with a CT integrated to the equipment (PET-CT). That technological advance brought an increase of specificity since it was possible to correlate the molecular findings with the exact anatomical location and structural abnormality seen on CT. That’s why the more recent systematic reviews evaluating the use of PET-CT in thyroid cancer showed better accuracy values as compared to “old” meta-analysis (7).

A very interesting paper published in this issue of *Archives of Endocrinology and Metabolism* (AE&M) by Yang and cols. (8) is the first series enrolling a reasonable number of Brazilian patients to be evaluated by PET-CT in detecting recurrent thyroid cancer. It is really an interesting contribution since it divided the patients in three different groups. The third group was the one where we wouldn’t expect great performance for PET-CT, as shown by the authors, since they included differentiated tumors with elevated serum Tg and positive WBS (2). The first group was divided in two, 1A (elevated Tg and negative conventional imaging and WBS) and 1B (elevated TG, and WBS not compatible with conventional imaging finding or level of Tg). Here we can make some comments. The greater impact would be detecting foci of recurrence where no other test is able to do. We would expect to see better incremental diagnostic value of PET-CT in those cases where no abnormalities are seen on US, CT or MRI. However, in that group the authors could only include nine patients, a very small number that precludes stronger conclusion regarding the value of the method tested in this article. Also the authors did not comment in that particular group how many patients had the PET-CT scans done under TSH stimulation or not. They stated that in significant percentage of patients it was not applied TSH stimulation for the PET scans. It is true that it is controversial the value of TSH stimulation in increasing the accuracy of PET in thyroid cancer, however, there has been no enough evidence in the literature yet to rule out any value of that stimulation for difficult and small-size disease detection. So we could conclude that in this small group of patients the PET did not add any clinical value and in a worst scenario lead to unnecessary biopsy caused by false-positive findings in cervical lymph nodes in three patients.

In the group IB, many patients showed alterations on conventional imaging at the same location seen of FDG-PET. If the location was the same of FDG-PET, and not compatible with the WBS, could we assume that the CT or MRI finding was enough to confirm the recurrence? Moreover, it is very well know that CT has a better detection rate of lung metastasis compared to PET, since size of the nodule is a limitation factor for the resolution of the modern PET scanners (9). Even though the small nodules detected by CT might be unspecific or indeterminate only by anatomical analysis, under the circumstance of rising Tg and high-risk profile of the patient for recurrence, it would be fair to consider those findings at least suspicious for recurrent disease. Moreover, to be detected by PET, those findings in the lungs must be large or the lung nodules numerous enough to be depicted by PET. The authors stated very well the limitations of the study, and then, in this group 1B could the CT/MRI alone be effective enough to change the clinical management in certain number of those thirteen patients, with no incremental information provided by PET? The question regarding that comment concerns the cost-effectiveness of doing PET-CT in all patients, including those with abnormalities already seen on conventional structural cross-sectional imaging.

The same issues can be discussed for the Group 2. Many patients showed abnormalities on lungs by CT and also by PET. Has PET also provided real incremental value towards the CT findings or just confirmed the CT abnormalities as related to the thyroid cancer recurrence?

Another limitation is the small number of patients regarding each group with unfavorable histology, a limitation that is expected considering the low prevalence of those histological types. There is no data in the literature evaluating the GLUT and HK-II expression in the various aggressive histology thyroid tumors. So, some conclusions must be taken with caution regarding the power of sample and considering some methodological aspects inherent to a retrospective study. Nevertheless, this is the first large cohort Brazilian study evaluating the clinical value of PET-CT in high-risk thyroid cancer patients, and hopefully will stimulate other groups to replicate that study to confirm the very interesting findings published in this issue of the AE&M.
